# Uptake and Immunomodulatory Properties of Betanin, Vulgaxanthin I and Indicaxanthin towards Caco-2 Intestinal Cells

**DOI:** 10.3390/antiox11081627

**Published:** 2022-08-22

**Authors:** Yunqing Wang, Ganwarige Sumali N. Fernando, Natalia N. Sergeeva, Nikolaos Vagkidis, Victor Chechik, Thuy Do, Lisa J. Marshall, Christine Boesch

**Affiliations:** 1 School of Food Science and Nutrition, Faculty of Environment, University of Leeds, Leeds LS2 9JT, UK; 2 School of Chemistry, Faculty of Engineering and Physical Sciences, University of Leeds, Leeds LS2 9JT, UK; 3 School of Design, Faculty of Art, Humanities and Cultures, University of Leeds, Leeds LS2 9JT, UK; 4 Department of Chemistry, University of York, York YO10 5DD, UK; 5 School of Dentistry, Faculty of Medicine and Health, University of Leeds, Leeds LS2 9LU, UK

**Keywords:** betalains, availability, intestinal uptake, inflammation, antioxidant, Caco-2 cells

## Abstract

The present study aimed to compare the absorption and transport patterns of three main betalains, betanin, vulgaxanthin I and indicaxanthin, into intestinal epithelial cells and to assess their distinct molecular effects on inflammatory and redox-related cell signalling in association with their radial scavenging potencies. All three betalains showed anti-inflammatory effects (5–80 μM), reflected by attenuated transcription of pro-inflammatory mediators such as cyclooxygenase-2 and inducible NO-synthase. Concomitant increases in antioxidant enzymes such as heme oxygenase-1 were only observed for betanin. Moreover, betanin uniquely demonstrated a potent dose-dependent radical scavenging activity in EPR and cell-based assays. Results also indicated overall low permeability for the three betalains with *P*_app_ of 4.2–8.9 × 10^−7^ cm s^−1^. Higher absorption intensities of vulgaxanthin and indicaxanthin may be attributed to smaller molecular sizes and greater lipophilicity. In conclusion, betanin, vulgaxanthin I and indicaxanthin have differentially contributed to lowering inflammatory markers and mitigating oxidative stress, implying the potential to ameliorate inflammatory intestinal disease. Compared with two betaxanthins, the greater efficacy of betanin in scavenging radical and promoting antioxidant response might, to some extent, compensate for its poorer absorption efficiency, as demonstrated by the Caco-2 cell model.

## 1. Introduction

**Figure 1:**
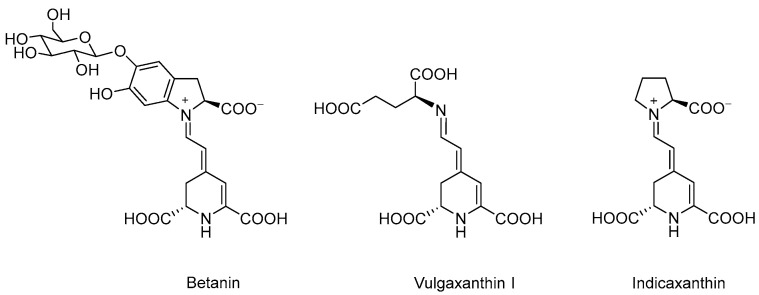
Chemical structures of betanin (BET), vulgaxanthin I (VUL) and indicaxanthin (IND).

Betalains are well known as food colourants, mainly sourced from plants of the *Caryophyllales* order, such as beet, cacti and amaranth. Approximately 75 members of the betalain family have been identified, with composition and concentration varying between plant species and varieties [1]. With core structures formed by the condensation between carbonyl and amine moieties (Figure 1), betalains are categorised into red-violet betacyanins (e.g., betanin, phyllocactin) and yellow-orange betaxanthins (e.g., vulgaxanthin, indicaxanthin). The molecules are susceptible to various types of decomposition, such as Schiff base hydrolysis and decarboxylation with their stability being maintained at pH 4–7, low temperature, darkness and absence of oxygen [2,3,4].

Radical scavenging and anti-inflammatory activity of some abundant betalains, such as betanin and indicaxanthin, are increasingly highlighted in the scientific literature [5,6,7,8]. The inflammatory response is largely driven by the transcription factor nuclear factor-κB (Nf-κB), involving the upregulation of pro-inflammatory cytokines and enzymes such as cyclooxygenase-2 (COX-2) and inducible nitric oxide synthase (iNOS) [9]. Tan et al. [10] have demonstrated alleviation of paraquat-induced renal inflammation following betanin administration to rats, attributed to the inhibition of NF-κB signalling. Allegra et al. [11] further reported strong suppression of COX-2 (by 88%) and iNOS (75%) following indicaxanthin treatment in a rat model of carrageenan-induced pleurisy. Meanwhile, betanin appeared to enhance the cellular defence system via interacting with nuclear factor-erythroid-2-related factor 2 (Nrf2) hence promoting the synthesis of antioxidant enzymes [12]. The subtle regulation of downstream targets of Nf-κB and Nrf2 signalling by specific betalains, therefore, revealed their influence on the cellular immune response and redox status. Additional evidence from in vivo animal and human studies mentioned the amelioration of lipid peroxidation, anti-hyperglycaemic, anti-tumour and hepato-/neuro-/cardio-protection [13,14,15], indicating the potential of betalains to deliver a range of health functions. However, most research in the literature focused on betanin and indicaxanthin, with other members of the betalain family, such as vulgaxanthin I, being scarcely explored.

Recent studies have reported the anti-inflammatory effect of polyphenols on inflamed intestinal Caco-2 cells via regulating the pro-inflammatory transcription factors and enzymes [16,17]. It implies the potential contribution of polyphenols in mitigating chronic inflammatory gut disorders, such as inflammatory bowel disease (IBD). IBD is characterised by the progressive disturbance of immune homeostasis at the intestinal epithelial level with higher susceptibility toward colorectal cancer [18]. Accordingly, it is also pivotal to improve the understanding of the inflammation and redox-related cellular effects of purified betalain molecules, which were investigated in this study on the stimulated intestinal epithelium.

Despite all the potential physiological effects, the bioavailability of betalains is considered a prerequisite to their overall health benefits in vivo. Bioavailability is commonly defined as the fraction of ingested component that enters the systemic circulation while maintaining the bioactivity at a specific site [19,20]. Pharmacokinetic and urinary excretion profiles from human and animal studies indicate inefficient uptake and low urinary excretion of betacyanins (ca. 3%) [5,21,22], as evidenced by the presence of native compounds and metabolites in physiological fluids [23,24]. The in vitro simulation of the human intestinal epithelial barrier is commonly utilised for monitoring the uptake and transport of drugs and nutrients. Using differentiated Caco-2 cells as a model, Tesoriere et al. [25] suggested paracellular passive diffusion as the predominant transport mechanism for betanin and indicaxanthin. Yet overall, the investigation of the availability of betalain compounds is still lagging behind compared to other bioactives such as anthocyanins [26,27,28].

The current research aimed to compare the potency of three abundant betalains—betanin (BET), vulgaxanthin I (VUL) and indicaxanthin (IND) towards radical scavenging, as well as attenuation of inflammation and oxidative stress in intestinal Caco-2 cells. Moreover, intracellular accumulation and transport kinetics of three betalains were investigated via the differentiated Caco-2 monolayer model.

## 2. Materials and Methods

### 2.1. Reagents, Materials and Samples

All reagents for cell culture (medium, buffer, antibiotics) were purchased from Gibco Cell Culture Products, Thermo Fischer Scientific (Loughborough, UK) unless specifically stated. As well, organic solvents used (HPLC and LC-MS grade) were purchased from Fisher Scientific. Chemicals including thiazolyl blue tetrazolium bromide (MTT), 2-(N-morpholino)ethanesulfonic acid (MES) and sulforhodamine B (SRB) were purchased from Merck Sigma Aldrich (Dorset, UK). All other reagents were acquired from Invitrogen™ Thermo Fisher Scientific, Bio-Rad (Watford, UK), Scientific Laboratory Supplies Ltd. (Nottingham, UK), VWR International Ltd. (Poole, UK) and Starlab UK Ltd. (Blakelands, UK).

The BET, VUL and IND pigments used were previously isolated from red and yellow beetroot (*Beta vulgaris* L.) and yellow prickly pear (*Opuntia ficus-indica* L.), respectively, using the flash column chromatography method as described in Fernando et al. [29]. The purities of previously isolated BET, VUL and IND have reached 97%, 79% and 95%, respectively, as determined by high-performance liquid chromatography with mass spectrometry (LC-MS).

### 2.2. Cell Culture and Maintenance

The Caco-2 cell line, derived from colorectal adenocarcinoma, was purchased from the European Collection of Authenticated Cell Culture (ECACC). Cells were cultivated in Dulbecco’s Modified Eagle Medium (DMEM) containing 4.5 mg mL^−1^ D-glucose and 0.11 mg mL^−1^ pyruvate, which was supplemented with 10% foetal bovine serum (FBS) (*v*/*v*), 1% non-essential amino acids (*v*/*v*), penicillin (50 U mL^−1^) and streptomycin (50 μg mL^−1^). The cells were kept under standard conditions (37 °C, 5% CO_2_). All experiments used Caco-2 cells between passage numbers 14 and 20.

### 2.3. Cytotoxicity Assay

After incubation with increasing concentrations of betalains (1–1000 µM) for 3–6 h, cells were rinsed with Dulbecco’s phosphate-buffered saline (DPBS) and exposed to MTT reagent (0.4 mM) dissolved in FBS-free DMEM for 3 h at 37 °C in the dark. After removal of medium and washing, cells were destained by DMSO, incubated on a plate shaker in the dark for 10–15 min and absorbance determined at 590 nm using Tecan Spark 10M^TM^. Cell viability was calculated as percentage of betalain-free medium control.

### 2.4. Expression of Pro-Inflammatory and Antioxidant Markers

Confluent Caco-2 cells were stimulated with a cocktail of cytokines comprised of tumour necrosis factor-α (TNF-α, 20 ng mL^−1^), interleukin-1β (IL-1β, 40 ng mL^−1^), interferon-γ (IFN-γ, 10 ng mL^−1^) and lipopolysaccharide (LPS, 100 ng mL^−1^) [30] in the presence or absence of BET, VUL and IND treatments (5–80 µM) for 6 h. With the completion of incubations, cells were washed with cold DPBS and lysed with TRIsure reagent (Scientific Laboratory Supplies Ltd.). Total RNA of cell samples was extracted in accordance with the manufacturer’s instructions. After resuspending the RNA pellets in DEPC water, concentration and quality were determined using the NanoQuant plate (Tecan Spark 10M plate reader) at 260 and 280 nm. Reverse transcription of RNA was achieved using the iScript™ cDNA Synthesis Kit (Bio-Rad). Transcription levels of target genes—interleukin 6 (IL-6), IL-8, COX-2, iNOS, NADPH oxidase 1 (NOX-1), NAD(P)H quinone dehydrogenase 1 (NQO-1), heme oxygenase 1 (HO-1), glutamate-cysteine ligase catalytic subunit (GCLC), glutathione peroxidase 1 (GPX-1), glutathione S-transferase 1 (GSTA-1) and GSTP-1 were evaluated using SybrGreen based reagent (SensiFast SYBR Green, SLS Ltd., Galveston, TX, USA) via real-time qPCR using StepOne Plus Real-Time PCR System (Applied Biosystems, Nottingham, UK). Primers of target genes were designed and blasted using the NCBI database, whose PCR product sizes were confirmed by 2% agarose gel electrophoresis. The list of primer sequences used in the study is presented in Supplemental Table S1. The calculation of target gene levels was based upon the Livak (2^−ΔΔCT^) method with normalisation to the housekeeping gene, β-actin [31]. Results were displayed as normalised gene levels relative to that of the stimulated control.

### 2.5. Quantification of Intracellular ROS Formation

The generation of reactive oxygen species (ROS) within Caco-2 cells was determined in 96-well plates with the probe 2′,7′-dichlorodihydrofluorescein diacetate (DCFH_2_-DA), which is intracellularly deacetylated and oxidised to highly fluorescent 2′,7′-dichlorofluorescein (DCF) [32,33]. DCFH_2_-DA (30 µM) dissolved in PBS with 1% FBS (*v*/*v*) was applied to the confluent Caco-2 cells and incubated for 40 min at 37 °C. After washing, cells were treated with BET, VUL and IND (5–80 μM) for 1 h and subsequently stressed with H_2_O_2_ (200 μM) for a further 1 h. The plate was scanned using Tecan Spark 10M^TM^ with excitation λ of 485 nm and emission λ of 535 nm. Subsequently, the protein content of each well was determined by SRB assay [33]. Briefly, the medium was removed and the cells were washed with PBS and submerged with 50 μL of 0.004% SRB solution (*w*/*v*) in 10% trichloroacetic acid (*w*/*v*). Cells were incubated at room temperature for 15 min and washed with 1% acetic acid (200 μL, *v*/*v*). After treating with Tris base (10 mM, 100 μL), cells were incubated for 5 min under shaking conditions. Finally, the plate was scanned using a Tecan plate reader with excitation λ of 540 nm and emission λ of 590 nm. Results of the ROS assay were normalised to the relative protein content for each well and expressed as the relative fluorescence intensity % (RFI) with respect to the value of positive control.

### 2.6. Radical Assays and Electron Paramagnetic Resonance (EPR)

Electron paramagnetic resonance (EPR) with spin trapping was used to determine superoxide and DPPH radical scavenging abilities of betalains reflected by the reduction of relative signal strength. EPR spectra were recorded 1 min after reagent mixing on a JEOL X320 spectrometer (X-band) in glass capillaries (0.8 mm ID). Superoxide assay was carried out by mixing the following solutions in 30% aqueous ethanol as solvent: hypoxanthine (1 mM, 55 μL), 5,5-dimethyl-1-pyrroline-N-oxide (DMPO) (1 M, 15 μL) and deferoxamine (10 mM, 10 μL). Individual betalains (200 and 500 μM) or solvent control were subsequently added in a volume of 10 μL. The reaction was initiated by the addition of 2 U mL^−1^ xanthine oxidase (10 μL), achieving 20 and 50 μM as final concentrations of betalains in the mixture.

The 2,2-diphenyl-1-(2,4,6-trinitrophenyl)hydrazyl (DPPH) assay was conducted by mixing ethanol (40 μL) with 30% aqueous ethanol solutions of DPPH (100 μM, 50 μL) and purified betalains (200 μM, 10 μL), providing the working concentration of betalains as 20 μM. In both assays, acquisition of EPR spectra was started 1 min after reagent mixing. To improve the accuracy of measurements, the signal intensity for the superoxide assay was determined in triplicate by fitting the experimental spectra to the simulated superoxide-DMPO adduct spectra.

### 2.7. Trans-Epithelial Transport and Intracellular Accumulation of Purified Betalains

Trans-epithelial transport of purified betalains (AP-to-BL) was investigated in differentiated Caco-2 cells in 12-well plate transwell inserts (PET membrane, 0.4 μm pore size, Sarstedt) at an initial density of 1 × 10^5^ cells cm^−2^. Complete medium was added in both apical (AP) and basolateral (BL) chambers and was replaced every 2–3 days. Cell membrane integrity was monitored weekly through measurement of trans-epithelial electrical resistance (TEER) across the growth area until full differentiation of the cells after 21 days. During the experiment, Hanks’ balanced salt solution (HBSS) in the apical compartment was adjusted to pH 6.5 with MES (2 mM), while basolateral pH remained at 7.5 [25]. Purified BET, VUL and IND (420–1000 μM) were supplied to the apical side of the membrane and incubated at 37 °C for up to 2 h. As a marker compound of paracellular transport, phenolsulfonaphtalein (5 mM) was also applied to parallel cell membranes in the apical chamber. The study also evaluated the permeability of compounds through the Caco-2 membrane with a loosened tight junction in parallel wells which was achieved by 5-min incubation of differentiated membrane in trypsin EDTA (0.05%, *v*/*v*). The basolateral solution was collected and replaced every 30 min until 2 h, under initial rate conditions. The betalain-containing solutions were processed and submitted to LC-MS analysis, while phenolsulfonaphtalein was quantified spectrophotometrically at λ of 430 nm. TEER values were monitored at the beginning and the end of the experiment to check the impact of compounds on monolayer integrity. The apparent permeability coefficients (*P*_app_) were calculated as per Equation (1), where V = volume of basolateral solution, A = cell growth area, C_0_ = initial concentration of apical betalain and dC/dt = steady-state flux across the monolayer.

(1) $$P_{\mathrm{app}}=\frac{1}{C_{0}\times A}\cdot \frac{V\times dC}{dt}$$

For accumulation experiments, differentiated Caco-2 cells were incubated with purified BET, VUL and IND (250 μM) for 2 h in HBSS. The cell layer was washed twice with DPBS prior to being scraped into methanol, and pigments were subsequently extracted for LC-MS analysis. The total protein content of lysed cells was determined by bicinchoninic acid (BCA) assay (Pierce™ Thermo Scientific) in accordance with the manufacturer’s instructions.

### 2.8. LC-MS Analysis

Betalain compounds from the cell culture samples were quantified and identified by an ultra-high-speed LC fitted with a UV-Vis detector (SPD-20A), followed by a single quadrupole MS with an electrospray ionisation source (ESI) (Shimazu, 2020). HPLC separation was performed by a reverse phase C18 column (100 mm × 2.1 mm I.D., 3.5 μm) at the temperature of 35 °C. The eluent gradient was established as a binary mobile phase, i.e., 2% HCOOH (*v*/*v*) as solvent A and LC-MS-grade MeOH as solvent B. With a constant flow rate (0.13 mL min^−1^), the proportion of solvent B inclined from 5% to 25% (0–9 min) and to 70% (9–12 min) before returning to the initial 5% (13–16 min). Betacyanins and betaxanthins were detected at the wavelengths of 536 nm and 486 nm, respectively, and their molecular weights were monitored by ESI-MS with positive ion mode. Compounds were identified based on the *m*/*z* values and quantified using standard curves with purified betalains, as shown in Supplemental Figure S1.

### 2.9. Statistics

Data analysis was performed using Excel and Graphpad Prism 9.0. The data are displayed as mean values with SEM from 3–4 independent experiments. One-way and two-way analysis of variance (ANOVA) were combined with the Dunnett post-hoc test to determine the existence of significant differences between specific datasets at a confidence interval of 95%. Physicochemical parameters of pigments were estimated using Chem3D and ChemDraw Ultra 14 with AutoDock.

## 3. Results and Discussion

### 3.1. Modulation of Pro-Inflammatory Cytokines by Betalains

This study has employed IL-1β combined with TNF-α as the major inducers for the acute inflammatory response in Caco-2 cells, which is of relevance for intestinal inflammatory conditions such as IBD. Upon stimulation by inflammatory triggers, phosphorylation of inhibitor subunit, IκBα, catalysed by IκB kinase, allows Nf-κB dimer accumulation in the nucleus and binding to respective DNA sequences. It initiates and maintains the synthesis and release of a range of pro-inflammatory and immunoregulatory factors, leading to the manifestation and amplification of gut inflammation [9,30,34].

Compared to the medium control, the generation of IL-6 and IL-8 was markedly augmented after 6 h exposure to the stimulatory cocktail of cytokines. This activation, however, could be strongly suppressed by co-treatment with curcumin (10 μM), a strong antioxidant and anti-inflammatory compound, which served as a positive control (not shown). Before treating Caco-2 cells with betalain compounds, their impact on cellular viability was evaluated by the MTT cytotoxicity assay. Cell viability remained above 90% after 6 h incubation with all betalains at the concentration range of 1–100 μM, suggesting a negligible negative impact of target compounds on the cells.

**Figure 2:**
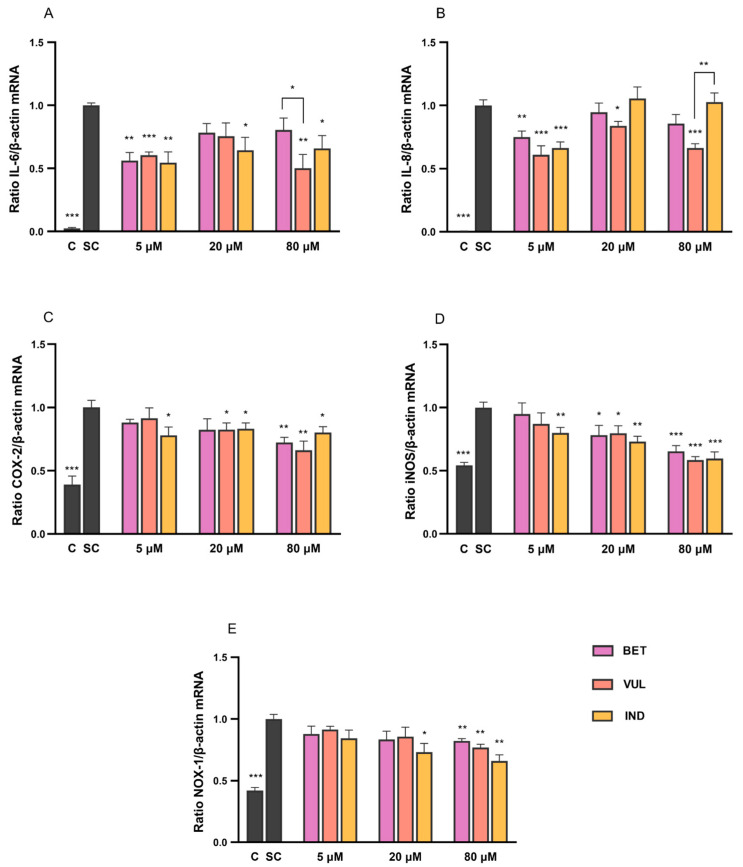
Effects of BET, VUL and IND (5, 20 and 80 µM) on mRNA levels of (**A**) IL-6, (**B**) IL-8, (**C**) COX-2, (**D**) iNOS and (**E**) NOX-1 following 6 h cytokine stimulation in Caco-2 cells. C and SC refer to negative medium control and cytokine-stimulated control, respectively. Data are mean with SEM of 3 replicates from independent cell experiments. Non-bridged asterisks indicate significant difference of treatment vs. SC: * *p* < 0.05, ** *p* < 0.01, *** *p* < 0.001.

As shown in Figure 2A,B, the incubation with BET, VUL and IND (5–80 μM) led to significant downregulation of IL-6 and IL-8 expressions by up to 50% and 39%, respectively, in comparison to cytokine control (SC). Results of IND were in partial agreement with Tesoriere et al. [35], albeit that the lack of dose-dependent response of cytokines plausibly indicated the distinct molecular interference at different betalain concentrations, which was also considered to be cell- and gene-specific. IND efficiently repressed IL-6 expression at all concentrations, while VUL was the most effective betalain to downregulate IL-8. All three betalains have potently inhibited the expression of both markers at 5 μM, whereas suppression of VUL appeared to be remarkably stronger at 80 μM compared to BET and IND (*p* < 0.05).

The participation of pro-inflammatory mediators, IL-6 and IL-8, is considered indispensable to the maintenance of intestinal homeostasis and immunologic function. They are commonly found overexpressed under gut inflammatory conditions such as Crohn’s disease [36]. As a cytokine, IL-6 contributes to the mucosal immune responses and is associated with bowel inflammation and colon necrosis. Meanwhile, the chemotactic IL-8 induces the recruitment of leucocytes in the inflamed region, followed by the secretion of surface antigens and reactive oxygen products [30,37,38]. The secretion of these two mediators is modulated by a cascade of intracellular signalling events, particularly with the stimulation of the NF-κB pathway in intestinal cells. This study indicated the downregulation of expression levels of IL-6 and IL-8 under inflammatory conditions, suggesting the capability of BET, VUL and IND to interfere with the NF-κB pathway in vitro hence their effectiveness in mitigating the inflammatory status of intestinal epithelium. However, the data related to VUL should be interpreted with caution, considering its relatively low purity (79%) compared to BET and IND (>90%), hence the possible contribution of impurities to the results.

### 3.2. Betalain Effects on Expression of NF-κB Target Enzymes

Unlike several constitutively expressed isozymes in the families of COX and NOS, COX-2 and iNOS are cytokine-inducible enzymes that are present at elevated levels under inflammatory conditions. COX-2 plays a critical role in regulating the serial reactions from arachidonic acid to prostaglandins and thromboxane, whose secretions are promoted considerably during the active phase of IBD and cause tissue oedema [39,40]. The enzyme iNOS catalyses the conversion from L-arginine to L-citrulline with the generation of nitric oxide (NO) as a byproduct. NOX-1 produces superoxide (O_2_^●−^) in the process of NADPH oxidation. Levels of NO and O_2_^●^^−^ that surpass the need for normal physiological activities generally lead to oxidative damage of tissue, inflammatory response and tumour development [41]. As target genes of the NF-κB signalling pathway, the expression of COX-2, iNOS and NOX-1 were effectively enhanced following the stimulation with IL-1β and TNF-α cytokines in this study.

As demonstrated in Figure 2C–E, co-treatment of stimulated cells with BET, VUL and IND (5–80 μM) has resulted in up to 34% attenuation of COX-2 and NOX-1 mRNA levels, with a greater impact on iNOS (<42% reduction). The three betalains have featured a dose-dependent inhibition for all target genes except IND to COX-2 expression which, given the absence of this effect on IL-6 and IL-8 expression, implied the modulation of pro-inflammatory markers by betalains to be gene-specific. At each concentration, the downregulation of individual markers was comparable among betalains. Results of anti-inflammatory properties of betalains were in agreement with other references that stated a marked inhibition of the protein levels and activities of COX-2 and iNOS enzymes by BET [42,43,44]. Tesoriere et al. [35] also provided evidence for IND (25 μM) as a potent suppressor of COX-2 protein levels (by 80%), iNOS (90%) and NOX-1 (89%) in a dose-dependent manner. In contrast, the attenuation effects of the three betalains on COX-2 and NOX-1 expression were relatively moderate in the present study, especially at 5 and 20 μM.

In addition, the two-factor ANOVA analysis suggested the similarity in the variation tendency of gene expression of COX-2, iNOS and NOX-1 as a function of betalain treatments (*p* > 0.05). This could be interpreted by the interplay between ROS/precursor and the COX-2 pathway which generally creates an inflammatory loop at the transcriptional level [39,45]. Consistent evidence was also found in studies in vitro and in vivo, indicating that excessively produced ROS and NO from NOX-1 and iNOS catalysis were often accompanied with enhancement of COX-2 activity hence downstream generation of prostaglandins [46,47,48]. Thus, the dose-dependent downregulation of COX-2 gene expression caused by betalains was akin to that of NOX-1 and iNOS genes, as shown in Figure 2.

### 3.3. Betalains Modulate the Expression of Nrf2 Target Enzymes

The electrophilic stress and cellular damage aggravated by the oxidant byproducts of inflammatory metabolism (e.g., ROS and NO) can promote Nrf2 signalling that orchestrates the synthesis of phase II antioxidant and detoxifying enzymes and facilitates the maintenance of cellular redox balance. In the presence of electrophiles or bioactive compounds, Nrf2 dissociates from the Kelch-like ECH-associated protein 1 (Keap1) and translocates to the nucleolus. Subsequent binding of Nrf2 to antioxidant-response-element (ARE) sequences in the promoter region of target genes leads to increased transcription of cell-protective proteins and enzymes such as NQO-1 [49,50].

NQO-1 in epithelial tissues majorly serves a role as reductase of quinones and superoxide derivatives in the redox cycle for stabilising radicals [51]. HO-1 enzyme is primarily produced to catalyse the degradation of toxic heme with co-functions of antioxidant, antiapoptosis and pro-angiogenesis [50,52]. The glutathione (GSH) system acts as an endogenous antioxidative defence in the intestinal epithelia against diet- and inflammation-induced ROS. A pivotal enzyme in GSH biosynthesis is glutamate-cysteine ligase, a strictly Nrf2 regulated enzyme with catalytic (GCLC) and modifier (GCLM) subunits [53].

GPXs and GSTs are also prominent contributors to the GSH system, with GPX isoenzymes catalysing H_2_O_2_ reduction coupled with GSH oxidation, while different GSTs assist in the process of chemical detoxification by conjugating reduced GSH to electrophiles such as carcinogens. Reported in previous studies, low expression and activity of GSTs in the human GI tract are often associated with colorectal cancer [50,54,55].

**Figure 3:**
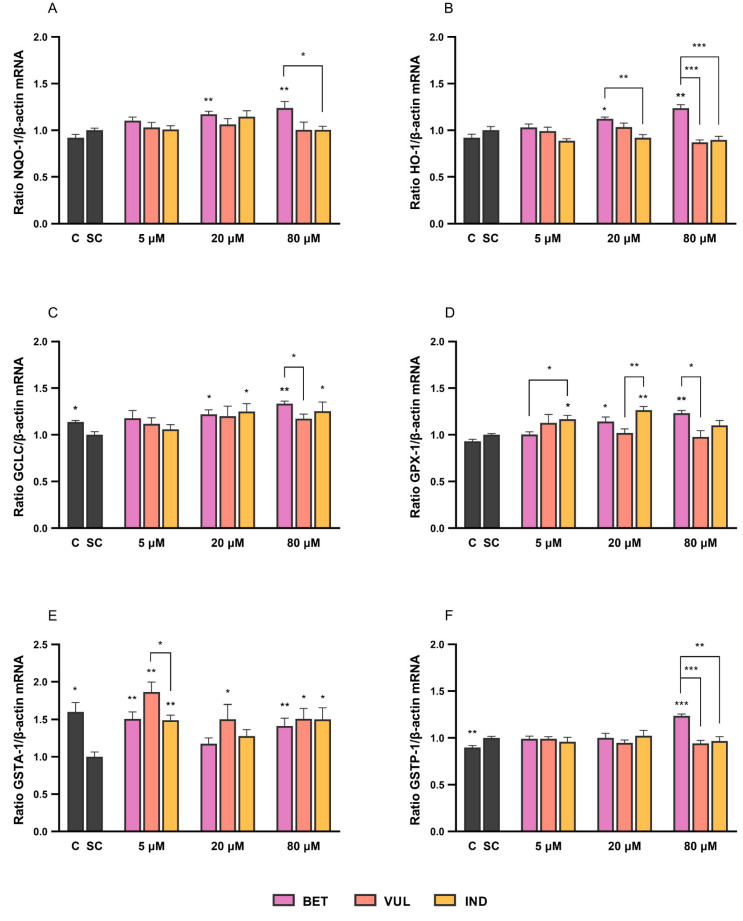
Effects of BET, VUL and IND (5, 20 and 80 µM) on mRNA levels of (**A**) NQO-1, (**B**) HO-1, (**C**) GCLC, (**D**) GPX-1, (**E**) GSTA-1 and (**F**) GSTP-1 following 6 h cytokine stimulation in Caco-2 cells. C and SC refer to negative medium control and cytokine-stimulated control, respectively. Data are mean with SEM of 3 replicates from independent cell experiments. Non-bridged asterisks indicate significant difference of treatment vs. SC: * *p* < 0.05, ** *p* < 0.01, *** *p* < 0.001.

As illustrated in Figure 3, BET has dose-dependently upregulated the expression of HO-1 (<24%), NQO-1 (<24%), GCLC (<33%), GPX-1 (<23%) and GSTP-1 (<24%). Meanwhile, IND led to an increment of GCLC (<25%) and GPX-1 (<26%). The modulation of GSTA-1 by betalains was more evident in comparison to GSTP-1. Even at concentration of 5 μM, all three compounds could compensate for the cytokine-induced downregulation of GSTA-1 (Figure 3E). When compared to BET (<51%) and IND (<50%), VUL generally showed more efficient induction of GSTA-1 (<86%) within the concentration range. Current results are partly in line with Esatbeyoglu et al. [56], who demonstrated moderate Nrf2 transactivation and augmented HO-1 protein levels in Huh7 cells, whereas Krajka-Kuzniak et al. [12] presented a dose-dependent increase in both mRNA levels and enzyme activities of NQO-1 and various GST enzymes in human hepatocytes following treatment with BET (2–20 μM). Nonetheless, the significant increases of NQO-1 and GSTM expression (ca. 60% and 100% increase in the baseline) at 20 μM BET in Kuzniak et al. [12] were not observed in the current study, which could be due to the cell-specific differences in responsiveness. With the exception of GSTA-1, the augmentation of selected targets, i.e., potential cytoprotective effects, was observed solely at 80 μM of BET.

### 3.4. Effects on Intracellular Oxidative Stress and Radical Scavenging Activities

**Figure 4:**
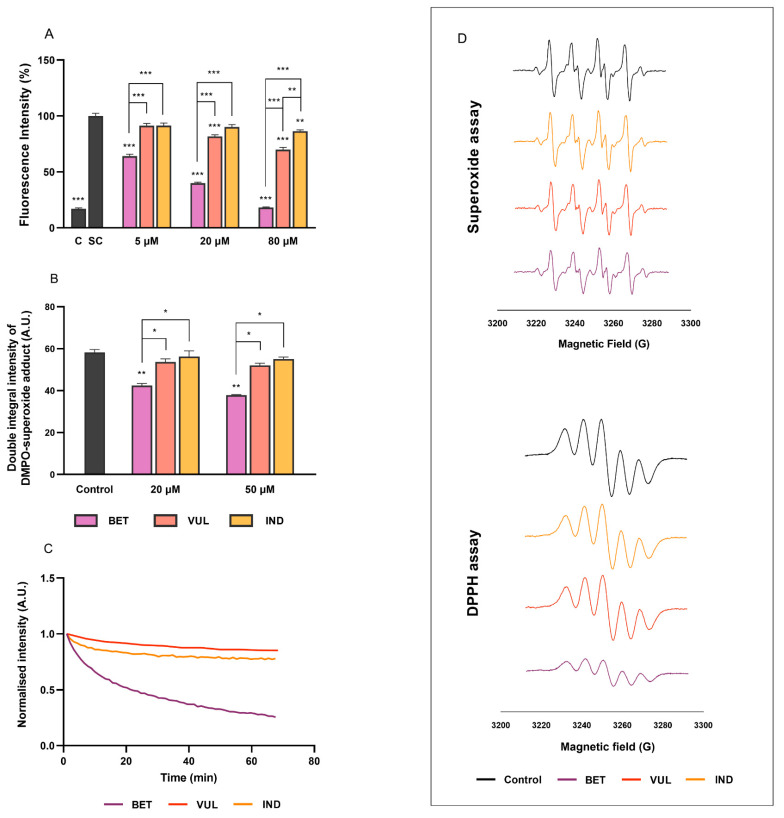
Radial scavenging capacity of betalains reflected by (**A**) intracellular ROS generation in Caco-2 cells, (**B**) double integral intensity of EPR signals of DMPO-superoxide adduct, (**C**) DPPH signal intensity in the presence of betalains, as well as (**D**) EPR spectra of superoxide and DPPH assays (post 30 min) in the absence (control) and in the presence of 20 μM purified betalains. C and SC refer to negative medium control and H_2_O_2_-stimulated control, respectively. Data in A and B are mean with SEM from three independent experiments. Non-bridged asterisks indicate significant difference of treatment vs. SC or Control: * *p* < 0.05, ** *p* < 0.01, *** *p* < 0.001.

The overexposure of the human intestine to ROS and toxic secondary metabolites is readily associated with increased cytokine production with subsequent impacts on DNA stability, resulting in cellular damage and inflamed tissues which may eventually lead to adenocarcinoma in the colon [55,57]. Mutually, intracellular ROS levels can be elevated via inflammatory and chemotactic induction, e.g., H_2_O_2_ in this study. BET, VUL and IND (5–80 μM) demonstrated potent radical scavenging capabilities by dose-dependent lowering of H_2_O_2_-stimulated ROS levels in Caco-2 cells (Figure 4A). The most pronounced suppression of ROS was exhibited by BET throughout the concentration range (<64%) compared to the two betaxanthins. Note that the magnitude of cellular ROS inhibition by BET in this study was found to resemble the properties of some well-known antioxidant flavonoids, e.g., quercetin and curcumin [32]. VUL displayed suppression of ROS generation only at 20 and 80 μM (<30%), more potent than IND which only showed a 14% reduction at 80 μM.

The direct radical scavenging capacities of all betalains were evaluated in the current study using EPR, with results presented in Figure 4B–D. Under spin-trapping conditions, BET (20, 50 μM) demonstrated high scavenging activity towards DPPH and O_2_^●^^−^ radicals. This was reflected by the significant decrease in EPR signal intensities in DPPH (<71%) and superoxide assays (<35%), respectively, hence lower concentrations of radical products. Our findings regarding BET were consistent with Esatbeyoglu et al. [56] who demonstrated dose-dependent radial quenching properties of BET in a similar concentration range (1–10 μM). In contrast to BET, VUL and IND did not exhibit any evident antiradical activity in both DPPH and superoxide assays, highlighting the different potencies of betaxanthins and betacyanins, which was in line with our previous findings [29].

In summary, the betalain-induced modulation of intracellular ROS production is likely a combination of different molecular events, including suppression of inflammatory signalling and activation of Nrf2-signaling, with the latter promoting transcription and synthesis of proteins and enzymes associated with cellular redox regulation, e.g., HO-1 and NQO-1. Down-regulation of targets driven by pro-inflammatory signalling, such as iNOS and NOX-1, may lead to reduced superoxide and subsequent radical generation in the cellular environment [55,58], thereby contributing to the redox balance. Further, the intrinsic reduction potential of betalains plays an essential role to radical scavenging properties which vary across different compounds following a structure–activity relationship [8]. The π-system resonance and multiple deprotonations of betalain structures under basic conditions render them more susceptible to electron donation, therefore radical stabilisation [59]. The stronger radical scavenging properties of BET compared to VUL and IND are possibly due to electron and proton transfer facilitated by phenol substituent in BET [60,61]. Meanwhile, in the case of VUL and IND, direct quenching of radicals was negligible, as observed from intracellular ROS and EPR data, whereas immunomodulation was considered the major contributor to their redox-regulating ability. According to the further results demonstrated in Section 3.5 and Section 3.6, betalains can be incorporated into and transported across the Caco-2 monolayer, during which they are likely involved in the modulation of cellular events.

### 3.5. Intracellular Uptake of Purified Betalains

The current study has further investigated the permeability of selected betalains to intestinal cells, determined through the uptake and transport characteristics. Uptake experiments using differentiated Caco-2 cells indicated the intracellular accumulation of three tested betalains after 2 h incubation (0.02 nmol of BET and 0.03 nmol of VUL and IND per mg of protein). Differentiated Caco-2 cells in wells with transwell insert served as a simulation of the compartmentalised environment of the small intestine. The incubation duration resembled the residence period of phytochemicals in the anterior segment of the human intestine and the prolonged incubation period did not cause differences in the amount of accumulated betalains. As aforementioned, the MTT assay indicated negligible cytotoxicity of tested betalain compounds (1–1000 μM) on the employed Caco-2 cell line within 3 h incubation (Supplemental Figure S2).

**Figure 5:**
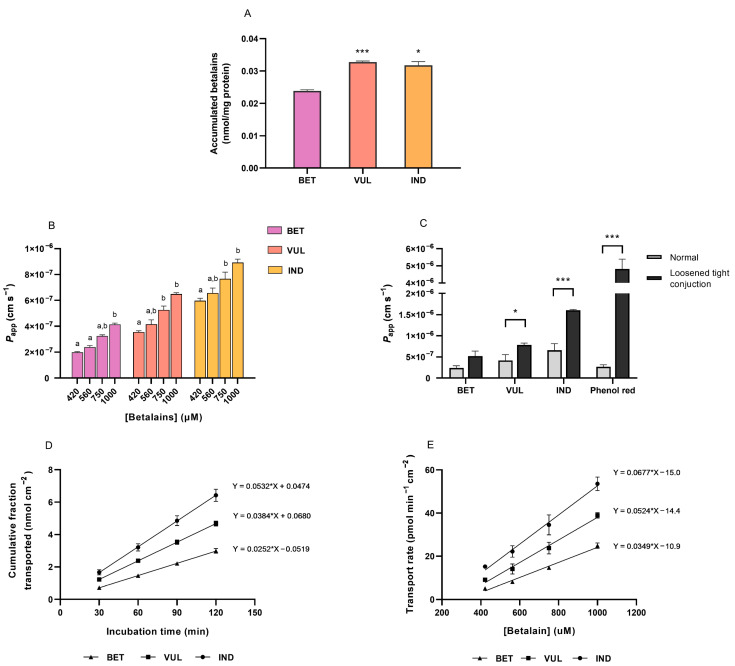
Uptake and trans-epithelial transport of purified BET, VUL and IND across Caco-2 intestinal membrane over 2 h incubation period, illustrated by (**A**) intracellular concentration, (**B**) apparent permeability coefficient (*P*_app_) at normal condition, (**C**) *P*_app_ across the membrane with normal vs. loosened tight junction, (**D**) transport kinetics at apical betalain concentration of 1 mM, and (**E**) dose-dependent transport rate of three betalains. Values are mean with SEM of 3–4 independent experiments. Significant differences are shown in (**A**,**C**) as * *p* < 0.05 and *** *p* < 0.001 comparing betalains to BET (**A**) and against controls (**C**). Different letters in (**B**) indicate significant differences between concentrations within one betalain (*p* < 0.05).

As shown in Figure 5A, the amounts of VUL and IND incorporated into Caco-2 cells were significantly higher compared to BET, whereas no difference was observed between absorptions of two betaxanthins. This divergence in cellular uptake can be attributed to the physicochemical characteristics of the phytochemical compounds [62]. The lipophilic core structure of betalains enabled a relatively low desolvation penalty of molecules when crossing a membrane [63]. As shown in Table 1, molecular size and weight incline following the sequence of IND, VUL and BET, which are accompanied by increasing counts of hydrogen bond donors and acceptors hence the polar surface area. These factors contribute positively to the water solubility of compounds yet have an inverse relationship with bioavailability, which will be discussed in more details in the following Section 3.6 on trans-epithelial transport.

The pKa values for carboxylates on the three betalains are proposed to be low (<4.5), especially for BET, which are on account of the vicinity of carboxylates to electron-withdrawing groups such as the positively charged iminium and delocalised π-system [25,64,65]. This helps interpret their susceptibility to deprotonation, hence multiple degrees of ionisation in the alkaline environment. The extent of interaction between betalains and lipid bilayer is governed by their lipophilicity and expressed as partition coefficient (Log *P*). Our data suggest that IND possesses relatively greater lipophilicity and lower susceptibility to carboxyl ionisation than VUL and BET in the environment of the small intestine (pH 6.5), which have, together with small molecular size, contributed to the relatively higher absorption of IND. This is also supported by results from previous studies [26,66].

### 3.6. Trans-Epithelial Transport of Purified Betalains

Transport kinetics (AP-to-BL) of purified BET, VUL and IND was monitored over the 2 h incubation period with concentrations ranging from 420–1000 μM, which are reasonable in reflecting a moderate dietary intake as well as in facilitating betalain quantification [24]. The TEER values, determined initially and post-incubation, did not differ, confirming the integrity of the Caco-2 monolayer after transport experiments.

The divergence in *P*_app_ among betalains was illustrated between different betalains along increasing concentrations (Figure 5B). After 2 h incubation with 1 mM individual betalain in the AP chamber, *P*_app_ values of BET, VUL and IND in the absorptive direction were obtained as 4.15 (±0.18) × 10^−7^ cm s^−1^, 6.49 (±0.21) × 10^−7^ cm s^−1^ and 8.93 (±0.52) × 10^−7^ cm s^−^^1^, respectively. Results of the study suggested the in vitro transport of three purified betalains to be on the lower end of the scale compared to several flavonoids and phenolic compounds whose *P*_app_ located between 2–8 × 10^−6^ cm s^−1^, e.g., quercetin, ferulic acid and gallic acid [26,62]. The poor permeability of three purified betalains was evidently boosted after loosening the tight junction of the Caco-2 membrane (Figure 5C), resembling the behaviour of phenolsulfonaphtalein as a paracellular indicator. Likewise, *P*_app_ values appeared to be notably lower than Tesoriere et al. [25], which might be attributable to the utilisation of higher pH at the AP chamber in this study, therefore possibly shifting physicochemical properties of pigments and their permeations from the reference. However, the experimental outcomes were consistent with preliminary tests, which assessed the influx transport of dextrin-coated BET standard (350–750 μM) in the 2 h window. The *P*_app_ measured (5.6–7.7 × 10^−8^ cm s^−1^) was roughly one-tenth of the values of purified BET, which is well conceivable that entanglement of polymeric dextrin at the surface of the Caco-2 membrane may have caused a physicochemical hindrance to the permeation of pigment.

Transport kinetics of tested betalains was demonstrated as a function of time and concentration in Figure 5D,E. The proportions of transported BET, VUL and IND within 2 h occupied 1.0%, 1.7% and 2.2% of total amounts applied, respectively, revealing the poor permeability of three betalains tested in the Caco-2 model. Regardless of concentrations, IND displayed the highest transport amount while BET showed the lowest (Figure 5D); the order was also observed in the *P*_app_ and transport rates of betalains (Figure 5B,E). Resembling the intracellular uptake, the physicochemical nature of individual betalains is reckoned as the determinant of their permeability [62]. Our in vitro data indicated higher cellular availability of IND compared to BET and VUL, which is in alignment with in vivo results demonstrating around 20-fold higher plasma concentration and urinary recovery of IND compared with BET. The considerable difference was likely due to the higher resistance of IND to digestive degradation, greater absorption efficiency and slower elimination rate [5,11,67]. The bioavailability of betalains is further heavily impacted by a variety of intrinsic as well as extrinsic factors.

The increasing linearity in Figure 5D has indicated a constant transport rate (AP-to-BL) of betalains over time at a specific concentration. The transport rate further revealed a first-order relationship with concentration, reflecting non-saturable kinetics of betalain permeation under experimental conditions (Figure 5E). During the transport process, there was no evidence of enzymatic modulation nor metabolic biotransformation, partially supporting the hypothesis regarding passive diffusion as the predominant mechanism of betalains crossing the intestinal epithelium [21,22,25].

For VUL and IND, the physicochemical parameters have fulfilled the “rule of 5” by Lipinski [68], indicating a relatively straightforward passage through the lipid membrane. Based on the phenolsulfonaphtalein result, diffusion of the two betaxanthins (MW < 600 Da) is likely to take place via the paracellular route with the generation of convective molecular flows through the aqueous intercellular space with rate-limiting tight junctions (i.e., pore pathway). The pores are formed in the complex of transmembrane proteins (e.g., claudin), whose size is believed to be inversely correlated to their quantity (i.e., pore theory) [69,70]. Therefore the permeation of small molecules is potentially facilitated by the more accessible transport passages due to the sieving effect of the paracellular network, which partly explained the higher availabilities of two betaxanthins compared with BET [71]. The possibility of transcellular diffusion, nevertheless, was not precluded [72]. In contrast, the greater size, higher structural complexity and hydrophilicity of BET presumably impede the process of crossing the hydrophobic bilayer region of Caco-2 cells; therefore, a limited absorption efficiency, dominated by paracellular diffusion is anticipated. More research is required to determine whether transport enzymes and carriers are involved in the uptake of betalains, particularly for glucosides of betacyanins that may share a comparable transport mechanism as those of polyphenols.

Overall, the availability experiments have demonstrated a low absorbability and permeability of betalains in the cell culture model, implying a low oral bioavailability in humans. Future research may focus on the investigation of transport mechanisms considering more physiological in vitro cell models, e.g., the employment of a mucus barrier to provide a better simulation of the human body [20].

### 3.7. Availability of Betalains, the Bottleneck to Immunomodulation

The anti-inflammatory and antioxidant features of tested betalains indicated their potential to prevent or mitigate intestinal inflammation diseases with cellular exposure to a concentration range of 5–80 µM. A comparable molecular effect was observed with several polyphenols (e.g., cyanidin-3-glucoside) and carotenoids (e.g., lycopene). For instance, in vitro studies have documented the anthocyanin-mediated downregulation (40–50 µM) of apical IL-6 (70–90% reduction) and tripled HO-1 expression in an inflamed Caco-2 cell model. While a further in vivo study showed the alleviation of colitis in mice after anthocyanin administration [36,73,74,75]. There was also evidence of the synergistic cytoprotective impact of coexisting bioactives (e.g., anthocyanin and lycopene) in light of immunoregulation and scavenging radicals [76]. Noteworthy, the dietary matrix of beetroot is a rich source of betalains, phenolics and nitrates, in which the constructive and/or destructive interference of components to their biofunctions can be speculated.

However, the results of the present study have provided an insight into the low transport efficacies of BET, VUL and IND being assimilated into the circulatory system despite the limitations of the Caco-2 cell model as an in vitro simulation. Current results are aligned with those of Sawicki et al. [24] and Wiczkowski et al. [23], who described low plasma concentrations and urinary recovery fractions in vivo following consumption of a betalain-rich diet. Apart from the restricted kinetics of trans-epithelial transport, betalain uptake has also been shown to be influenced by other factors, including pigment retention by food matrix and digestive decomposition [77]. Tesoriere et al. [78] illustrated various and significant degrees of loss (up to 70%) of purified BET and VUL, whereas minor loss of IND occurred along the digestion. Thus, the lower availability of BET and VUL is considered a bottleneck for their potential health effectiveness regardless of their promising bioactivities. Data regarding cellular effects of betalains in immune and endothelial cells [44,79] need to be interpreted in view of low availability and, therefore, low circulating betalain concentrations.

## 4. Conclusions

Bioactivity and cellular properties of betalains are less extensively explored in comparison to other phytochemicals, which is partially due to lacking availability of purified compounds. The current study demonstrated poor availability for the three isolated betalains: BET, VUL and IND. In a dose-dependent manner, the experiments illustrated a higher rate of intracellular uptake and trans-epithelial transport of betaxanthins compared with BET. This divergence is greatly attributed to the physicochemical features of different betalain molecules. Transport experiments also demonstrated first-order kinetics and the absence of betalain biotransformation, which partially supported the hypothesis that passive diffusion is adopted by betalain compounds as a predominant transport route. Regarding the cellular effects, betalains were found effective in mitigating inflammation as well as oxidative stress in Caco-2 cells. Whilst downregulation of inflammatory gene expression was evident for all betalains, BET was more potent in augmenting levels of redox-regulated phase II enzymes in the Nrf2 pathway and exerted stronger inhibition of radical concentration and intracellular ROS level, thus ameliorating oxidative damage of Caco-2 cells. The results suggest a positive role for betalains in alleviating inflammatory gut conditions; however, further research is warranted to establish the efficacy of betalains in vivo.

## Figures and Tables

**Table 1:** Physicochemical properties of selected betalains. Parameters include molecular weight (MW), number of hydrogen bond donors (HBD) and hydrogen bond acceptors (HBA), topological polar surface area (tPSA) and octanol/water partition coefficient (Log *P*). Data were acquired from ChemDraw 3D.

Betalains	MW (g mol^−1^)	HBD	HBA	tPSA (Å^2^)	Log *P*
BET	551.48	9	14	246.55	−2.50
VUL	340.29	5	9	173.59	−1.46
IND	309.3	4	7	126.94	−1.12

## Data Availability

Data is contained within the article and supplementary material.

## Supplementary Materials

The supporting information below can be downloaded at: https://www.mdpi.com/article/10.3390/antiox11081627/s1, Table S1. Human primer sequence information. Figure S1. Spectrophotometric chromatogram of purified betalain standards at the detection wavelengths of 536 nm and 486 nm. Individual peaks refer to vulgaxanthin I (1), indicaxanthin (2), betanin (3) and isobetanin (3’). Figure S2. Viability of Caco-2 cells (%) using MTT assay after treatment with increasing concentrations of individual betalains. Data are presented as mean with SEM of triplicates from independent cell passages.
