# Supplementary material for: Uptake and Immunomodulatory Properties of Betanin, Vulgaxanthin I and Indicaxanthin towards Caco-2 Intestinal Cells
Source: Antioxidants (Basel). 2022 Aug 22;11(8):1627. doi: 10.3390/antiox11081627 (PMC9405451; doi:10.3390/antiox11081627)
Supplement: Supplementary file 1 [file antioxidants-11-01627-s001.zip › antioxidants-1813650-supplementary.pdf]

Supplementry materials

Table S1. Human primer sequence information.

Markers	Forward sequence	Reverse sequence	Sequence ID
β-actin	5'- AGAGCTACGAGCTGCCTGAC -3'	5'- AGCACTGTGTTGGCGTACAG -3'	NM_001101.5
IL-6	5'- CGGCCACTCACCTCTTCAGAA-3'	5'- GGCAAGTCTCCTCATTGAATCC-3'	NM_001371096.1
IL-8	5'- TGGAGAAGTTTTTGAAGAGGGCT -3'	5'- CAACAGACCCACACAATACATGA -3'	NM_000584.4
COX-2	5'- ATTGACCAGAGCAGGCAGAT -3'	5'- CTCCACAGCATCGATGTCAC -3'	NM_000963.4
iNOS	5'- ACAGCACATTCAGATCCCCA -3'	5'- GCCGAGATTTGAGCCTCATG -3'	NM_000625.4
NOX-1	5'- GTCTCTTCCTCACCGGATGG -3'	5'- CCCACTACAGACTTGGGGTG -3'	NM_001271815.2
NQO-1	5'- AGAAAGGATGGGAGGTGGTG -3'	5'- ATATCACAAGGTCTGCGGCT -3'	NM_000903.3
HO-1	5'- CTTCTTCACCTTCCCCAACA -3'	5'- AGCTCCTGCAACTCCTCAAA -3'	NM_002133.3
GCLC	5'- CAATGGGAAGGAAGGTGTGT -3'	5'- GCGATAAACTCCCTCATCCA -3'	NM_001498.4
GPX-1	5'- CCGGGACTACACCCAGATGA -3'	5'- CTTGGCGTTCTCCTGATGCC -3'	NM_000581.4
GSTA-1	5'- CTTCTGCCCGTATGTCCACC -3'	5'- ACCAGATGAATGTCAGCCCG -3'	NM_001319059.2
GSTP-1	5'- GCGGGCAAGGATGACTATGT -3'	5'- GTCAGCGAAGGAGATCTGGTC -3'	NM_000852.4

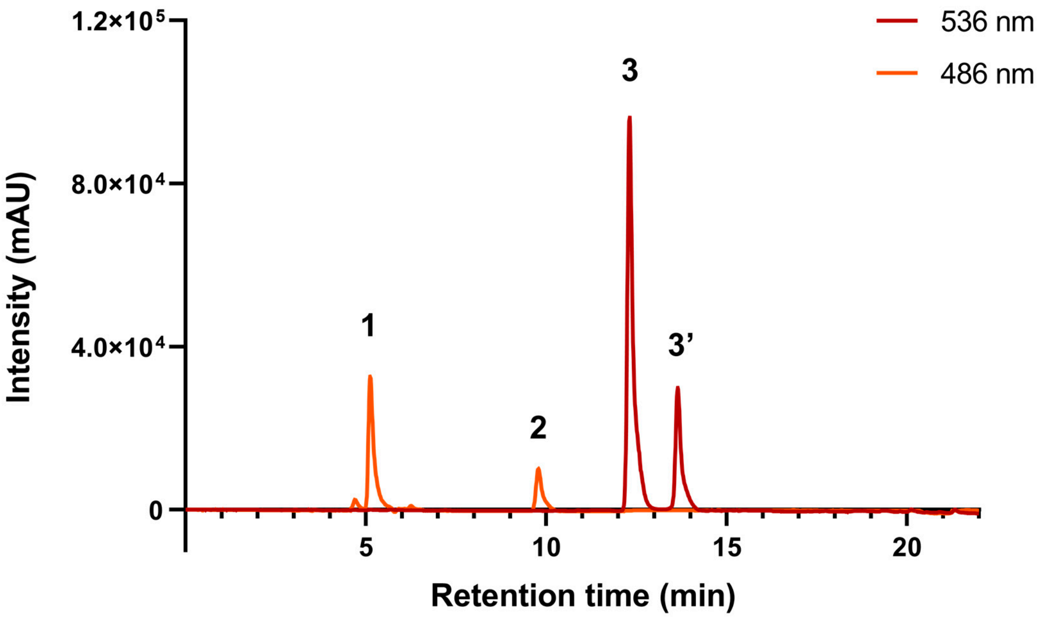

Figure S1. Spectrophotometric chromatogram of purified betalain standards at the detection wavelengths of 536 nm and 486 nm. Individual peaks refer to vulgaxanthin I (1), indicaxanthin (2), betanin (3) and isobetanin (3').

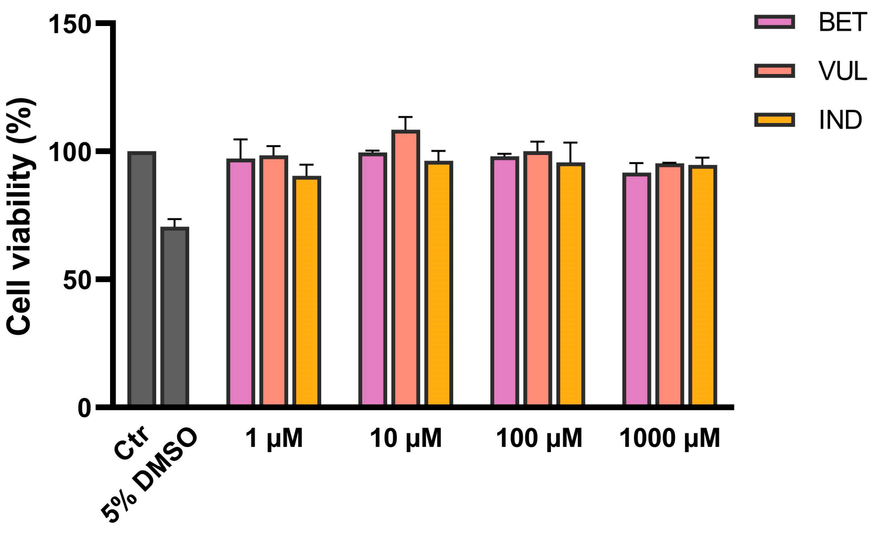

Figure S2. Viability of Caco-2 cells (%) using MTT assay after treatment with increasing concentrations of individual betalains. Data are presented as mean with SEM of triplicates from independent cell passag
